# Scalp metastasis from gastric cancer: A case report and literature review

**DOI:** 10.3892/ol.2014.2708

**Published:** 2014-11-19

**Authors:** CHUNXIA DU, RUOXI HONG, YUEHUA LIU, JINWAN WANG, HONGGANG ZHANG, XIAODUO YU

**Affiliations:** 1 Department of Medical Oncology, Cancer Institute/Hospital, Chinese Academy of Medical Sciences, Beijing 100021, P.R. China; 2 Department of Dermatology, Peking Union Medical College Hospital, Chinese Academy of Medical Sciences, Beijing 100730, P.R. China; 3 Department of Radiology, Cancer Institute/Hospital, Chinese Academy of Medical Sciences, Beijing 100021, P.R. China

**Keywords:** scalp metastasis, gastric cancer

## Abstract

The current report presents an extremely rare case of a 41-year-old female with advanced gastric cancer who developed scalp metastasis during the period of systemic chemotherapy. The patient did not exhibit any rash or plaque at the initial physical examination. Following the 11th cycle of chemotherapy, the patient complained of pain on the scalp and a pink lesion was identified in the parietal region on physical examination, which increased in size and became darker and ulcerated. Pathological biopsy of the lesion and cranial magnetic resonance imaging confirmed the diagnosis of scalp metastasis. The patient succumbed to the disease one month later. The English literature was searched in the PubMed database and four cases of gastric cancer metastatic to the scalp were found. The present report discusses the common clinical presentations of these four cases in combination with the current case.

## Introduction

Cutaneous metastasis from internal malignancy is relatively uncommon, with a reported frequency varying between 0.7% and 9%, among which breast, lung, oral mucosa and colorectal cancer are most likely to metastasize to the skin (1). Cutaneous involvement from gastric carcinoma is even rarer (1–3) and usually arises in the vicinity of the primary tumor (such as the abdominal wall) as non-specific nodules (4).

The scalp is an unusual site of cutaneous metastasis. Brownstein and Helwig previously reported that scalp metastasis accounts for 4% of all skin metastases (5). Gastric cancer metastatic to the scalp is extremely rare with few cases reported to date (6–9). The current report presents a case of scalp metastasis from gastric cancer and a review of the related literature in order to provide new insights into the diagnosis, treatment and prognosis of such cases in future. Patient provided written informed consent.

## Case report

**Figure 1:**
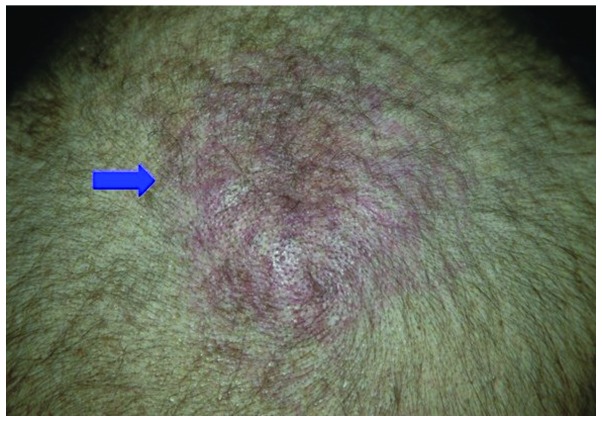
Image captured on December 9, 2010 shows the pink swollen lesion (3×3 cm^2^) over the parietal region.

**Figure 2:**
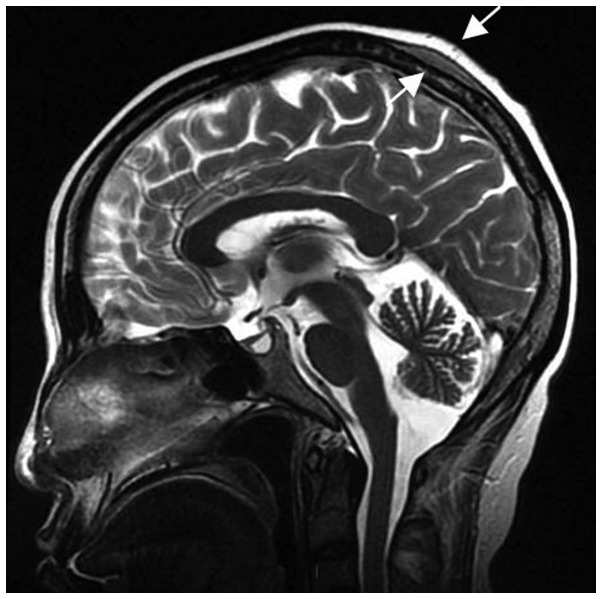
Midline sagittal T2-weighted image captured on December 10, 2010 shows localized thickening of the subparietal galea aponeurotica with homogeneously slightly high signal intensity, as indicated by the white arrow.

**Figure 3:**
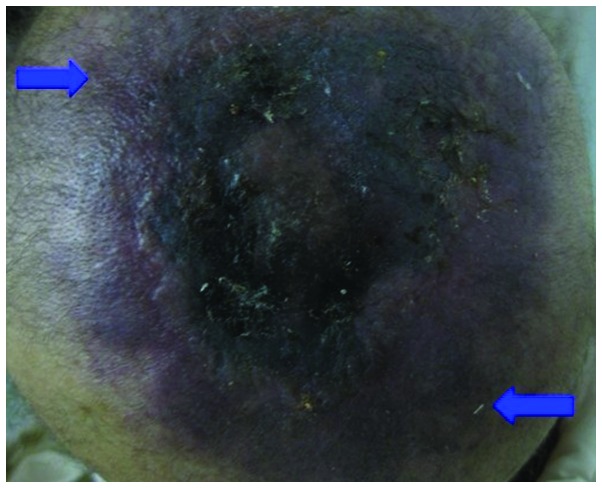
Image captured on January 17, 2011 shows that the scalp lesion (edges of the scalp lesion are indicated by the arrows) had increased in size (13×12 cm^2^) and become darker and ulcerated.

**Figure 4:**
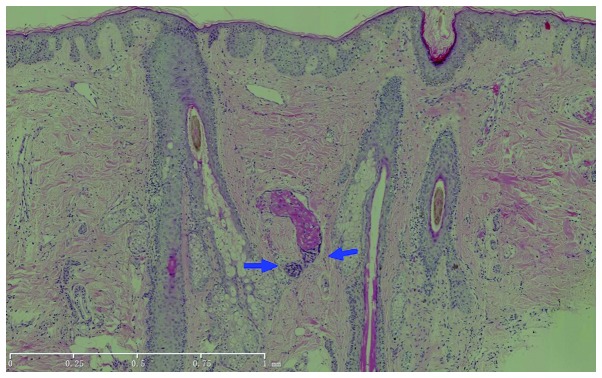
Pathological biopsy performed on January 19, 2011 identifies tumor emboli in the small vessels (as indicated by the arrows). Magnification, ×100.

**Figure 5:**
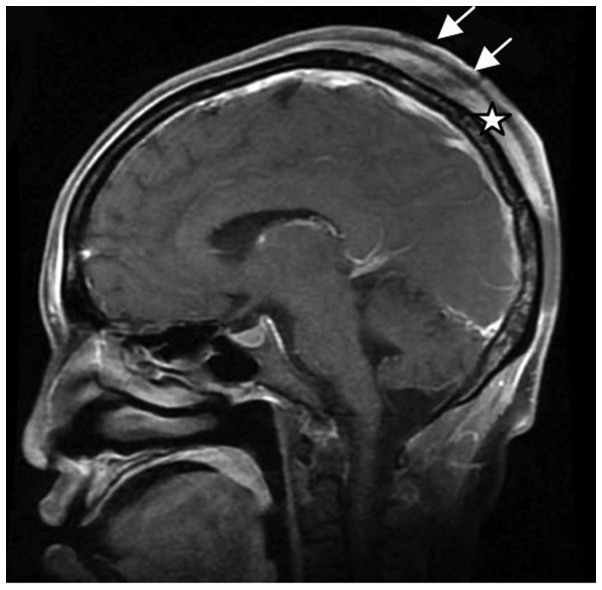
Midline sagittal T1-weighted image with fat-suppression following administration of contrast agents, captured on January 24, 2011. Image shows heterogeneous thickening of the galea aponeurotica, identified by the star and of the skin on the top of the brain with significant enhancement, as well as the dura mater of the parietal and occipital lobe. Defects are evident in the skin and are indicated by the white arrows. The diploë on the parietal and occipital bone is also shown to be heterogeneously enhanced.

A 41-year-old female patient was admitted to the Department of Medical Oncology, Cancer Institute/Hospital, Chinese Academy of Medical Sciences (Beijing, China) on July 21, 2010 due to complaints of upper abdominal pain for 10 months and lower back pain for three months. The patient’s Karnofsky Performance Status score was 90. No skin rash or plaque was observed on general physical examination. Multiple enlarged lymph nodes were palpable in bilateral cervical and supraclavicular regions, and chest palpation revealed tenderness over the seventh right rib. The abdomen was soft without palpable organomegaly. No point tenderness was identified under the xiphoid upon palpitation without muscle guarding or rebound tenderness. Complete blood count showed anemia (hemoglobin levels, 102 g/l), biochemistry tests were within the normal ranges and certain serum biomarker levels were elevated (CA19-9, 156.4 U/ml; CA72-4, 1,292 U/ml; CEA, within the normal range). Gastroscopy revealed a 1.0×1.2-cm submucosal lesion along the greater curvature of the gastric body. Pathological biopsy of the gastric lesion showed signet ring cell carcinoma and HER-2 staining was negative in tumor cells. Pathological biopsy of the supraclavicular lymph nodes showed metastatic carcinoma. Computed tomography (CT) scan from the neck to the pelvis revealed enlarged lymph nodes in the cervical, supraclavicular, mediastinal, hilar, perigastric and retroperitoneal regions, in addition to thickening of the gastric wall, bilateral ovarian metastases, pericardial effusion, bilateral pleural effusion and ascites. Radionuclide bone scan showed multiple bone metastases. Based on the previously described observations, the diagnosis of stage IV gastric signet ring cell carcinoma was determined. Between July 2010 and December 2010, the patient received 11 cycles of systemic chemotherapy using docetaxel (40 mg/m^2^d1), oxaliplatin (85 mg/m^2^d^2^) and 5-fluorouracil (400 mg/m^2^ bolus on days two and three plus 600 mg/m^2^ continuous intravenous infusion over 22 h on day one, twice every two weeks). During the interval of the second cycle of chemotherapy, the patient received local radiotherapy to the rib metastatic site due to unrelieved pains. The adverse effects of the chemotherapy included grade II gastrointestinal reactions, grade II thrombocytopenia and grade III neutropenia. Following four cycles of chemotherapy, the patient achieved partial response according to the RECIST guidelines (version 1.1) (10) and the results were confirmed following eight cycles. In early December 2010, the patient complained of pain in the scalp. Physical examination revealed a pink lesion measuring 3×3 cm^2^ on the scalp over the parietal region, with slight tenderness (Fig. 1). Further inquiry into the patient’s past history indicated a similar ‘skin disease’ at the same site several years previously, which had been cured by specific dermatologic drugs. Plain skull magnetic resonance imaging (MRI) scan showed local thickening of the subparietal galea aponeurotica (Fig. 2). The patient refused further examination. The patient’s follow-up at our department for regular chemotherapy found that the scalp lesion had increased in size (12×13 cm^2^) and become darker and ulcerated (Fig. 3). Therefore, the patient was referred to the dermatology clinic. Pathological biopsy of the lesion revealed tumor emboli in small vessels (Fig. 4). On January 21, 2011, the patient complained of sickness, vertigo and diplopia. The patient was admitted to our emergency room on January 24, 2011, and cranial MRI revealed scalp and dural metastases (Fig. 5). The patient was treated with mannitol and prednisolone to control intracranial hypertension. However, the symptoms were uncontrollable and the patient succumbed to the disease two days later.

We also performed a review of the associated literature. PubMed was searched using the key words ‘gastric cancer’, ‘cutaneous metastasis’ and ‘scalp’. The reference articles of the search results were screened and five case reports were found concerning scalp metastasis from gastric cancer, one of which was written in Spanish without availability of the English abstract and, therefore, was excluded from the study (Table I).

## Discussion

Scalp metastasis is a rare occurrence in <2% of patients with malignant metastases. Lung cancer (23.53%) has been recognized as the primary tumor most frequently metastasizing to the scalp, followed by colorectal (11.76%), liver (7.84%) and breast (7.84%) cancer. Notably, metastatic tumors of undetermined origin accounted for 29.41% of all metastatic scalp tumors (11).

Scalp metastatic lesions may grow unnoticed for a long period of time, manifesting as atypical nodules or plaques, or alopecia neoplastica in even rarer cases (8). As scalp metastasis lacks characteristic clinical presentations, it is often overlooked as an ordinary skin disease, which is the main reason for delayed diagnosis and treatment. According to the previous literature, a number of patients remain undiagnosed until 4–10 months following the identification of scalp lesions (6–8). In the present case report, the diagnosis of the scalp lesion was delayed for several weeks due to the confusing history of ‘skin disease’ and incompliance of the patient. Therefore, it is necessary to establish a full evaluation of any cutaneous lesion in patients with internal malignancies, and pathological biopsy is recommended.

Cutaneous metastasis portends a poor prognosis. The mean survival for patients with cutaneous metastasis ranges between one and 34 months depending on the tumor type (2). The majority of patients with cutaneous metastasis from primary lung, cervical or esophageal cancer succumb to their diseases three months after the development of cutaneous metastases. In three of the four previously reported cases of gastric cancer metastatic to the skin, the mean survival time was one month (2), while the survival time of the other patient remains unknown due to loss to follow-up. Similarly, the median survival time was two months in the three patients with gastric cancer following diagnosis of scalp metastases (as shown in Table I).

Cutaneous metastatic lesions often occur in the final stage of cancer, indicating that underlying cancer has spread extensively. The majority of patients exhibit concomitant metastases to other organs. Extensive radiological evaluations, including CT, MRI and positron emission tomography-CT, may provide more valuable information. Systemic chemotherapy is recommended as the major treatment (2,3). Previously, Frey *et al* reported a patient with cutaneous metastasis from gastric cancer who responded well to systemic chemotherapy and survived for >12 months (6). In cases where scalp lesions induce uncontrolled symptoms, such as pain, or appear as the only metastatic site, local excision/radiotherapy may be considered. However, no significant change in survival has been previously reported with any particular treatment available.

The present case report and review of the literature demonstrated the rarity of scalp metastasis in gastric cancer. Although cutaneous lesions usually reflect a more widely spreading disease, they may also present as the first sign of cancer. For patients with known internal malignancies, skin lesions often require further evaluation. Pathological biopsy must be performed when necessary. Extensive evaluations, including general physical examination and further radiological examination, are important for such patients. Scalp metastasis often indicates a grave prognosis with a mean survival time of less than three months. Systemic chemotherapy is the major treatment, but no particular treatment has been previously reported to change survival rates.

## Figures and Tables

**Table I:** Scalp metastasis from gastric carcinoma: review of the literature.

First author (year) [ref]	Age, years	Gender	First sign of gastric cancer	Site of cutaneous metastasis	Synchronous metastases	Time between scalp lesion identification and diagnosis, months	Time between diagnosis of scalp metastasis and mortality, months	Therapy following diagnosis of scalp metastasis	Response to therapy
Sakaki (1979) [9]	53	Female	N	Scalp	Dural and lymph nodes	0	1	Surgery	Patient succumbed to the disease 5 days following surgery
Kim (1999) [8]	36	Female	N	Scalp	Pelvis	10	2	No	-
Lifshitz (2005) [7]	73	Male	N	Upper forehead and scalp	Not found	4	7	Localized IL-2 treatment of the scalp lesion and radiotherapy	The plaques decreased in size following radiotherapy
Frey (2009) [6]	54	Male	Y	Scalp	Lung, liver and lymph nodes	4	>12^a^	Chemotherapy (docetaxel, cisplatin and 5-fluorouracil)	Scalp nodules disappeared and the primary tumor regressed
Present case (2011)	41	Female	N	Scalp	Lung, bone and lymph nodes	1	1	No	-

a Patient’s progression-free and overall survival were not reported in the literature, the author was contacted and the information was obtained that the patient responded to chemotherapy and was recurrence-free for ≥12 months.

N, no; Y, yes; IL-2, interleukin 2.
